# Sotos syndrome

**DOI:** 10.1186/1750-1172-2-36

**Published:** 2007-09-07

**Authors:** Geneviève Baujat, Valérie Cormier-Daire

**Affiliations:** 1 Department of Medical Genetic, Hospital Necker-Enfants Malades, 149 rue de Sèvres, 75743 Paris Cedex 15, France

## Abstract

Sotos syndrome is an overgrowth condition characterized by cardinal features including excessive growth during childhood, macrocephaly, distinctive facial gestalt and various degrees of learning difficulty, and associated with variable minor features. The exact prevalence remains unknown but hundreds of cases have been reported. The diagnosis is usually suspected after birth because of excessive height and occipitofrontal circumference (OFC), advanced bone age, neonatal complications including hypotonia and feeding difficulties, and facial gestalt. Other inconstant clinical abnormalities include scoliosis, cardiac and genitourinary anomalies, seizures and brisk deep tendon reflexes. Variable delays in cognitive and motor development are also observed. The syndrome may also be associated with an increased risk of tumors. Mutations and deletions of the *NSD1 *gene (located at chromosome 5q35 and coding for a histone methyltransferase implicated in transcriptional regulation) are responsible for more than 75% of cases. FISH analysis, MLPA or multiplex quantitative PCR allow the detection of total/partial *NSD1 *deletions, and direct sequencing allows detection of *NSD1 *mutations. The large majority of *NSD1 *abnormalities occur *de novo *and there are very few familial cases. Although most cases are sporadic, several reports of autosomal dominant inheritance have been described. Germline mosaicism has never been reported and the recurrence risk for normal parents is very low (<1%). The main differential diagnoses are Weaver syndrome, Beckwith-Wiedeman syndrome, Fragile X syndrome, Simpson-Golabi-Behmel syndrome and 22qter deletion syndrome. Management is multidisciplinary. During the neonatal period, therapies are mostly symptomatic, including phototherapy in case of jaundice, treatment of the feeding difficulties and gastroesophageal reflux, and detection and treatment of hypoglycemia. General pediatric follow-up is important during the first years of life to allow detection and management of clinical complications such as scoliosis and febrile seizures. An adequate psychological and educational program with speech therapy and motor stimulation plays an important role in the global development of the patients. Final body height is difficult to predict but growth tends to normalize after puberty.

## Disease name and synonyms

Sotos syndrome (OMIM 117550)

Cerebral Gigantism syndrome

## Definition

Sotos syndrome is a childhood overgrowth condition, first described in 1964 by Sotos *et al *[1], though the first patient described may have been reported in 1931 [2]. The four major diagnostic criteria were established in 1994 by Cole and Hughes [3], based on the systematic assessment of 41 typical cases: overgrowth with advanced bone age, macrocephaly, characteristic facial appearance, and learning difficulties. These clinical criteria remained the cornerstone for the diagnosis of Sotos until 2002. The recent identification of *NSD1 *mutations and deletions [4] has allowed re-evaluation of the features of this condition [5-9]. In 2005, Tatton-Brown and the Childhood Overgrowth Collaboration Consortium reviewed the clinical features of 239 Sotos cases with *NSD1 *abnormalities. They confirmed that overgrowth (including height and occipito-frontal circumference), dysmorphism and learning disability were present in 90% of these *NSD1*-positive individuals, with a wide spectrum of associated features including macrocephaly, advanced bone age, neonatal jaundice and hypotonia, seizures, scoliosis, cardiac defects and genitourinary anomalies [10].

## Epidemiology

Sotos syndrome is probably one of the most common overgrowth conditions, after the Beckwith-Wiedemann syndrome. The exact birth prevalence remains unknown. Hundreds of cases are reported. The literature on Sotos syndrome, before the identification of the *NSD1 *gene responsibility, has to be read carefully since it is mixed with publications that evidently do not deal with the Sotos syndrome.

## Clinical description

For forty years, the diagnosis of Sotos syndrome has been based on the subjective evaluation of clinical features. However, although the combination of craniofacial features may be distinctive, the other components are non-specific. This lack of specific clinical features was often the center of nosologic discussion [3,11]. Recently, Tatton-Brown and Rahman analyzed helpfully the clinical features of a large series of more that two hundred Sotos syndrome cases with proven abnormalities in *NSD1 *[10,12].

Generally, pregnancy is described as normal; however, toxemia or pre-eclampsia is mentioned in several cases [11]. Mean gestational age is normal (39 weeks) but the infants are large for their gestational age. According to Cole and Hughes [3], birth length, weight, and occipito-frontal circumference (OFC) are respectively 3.2, 1.0, and 1.8 standard deviations above the mean. Length is likely more increased than weight. Indeed, the birth weight of 76% of the 107 Sotos cases reported by Tatton-Brown was less than the 98^th ^centile and the mean birth weight centile was 75^th^-91^st ^[12]. In the neonatal period, many infants experience early feeding difficulties (40% required tube feeding [3]), variable degree of congenital hypotonia, jaundice by hyperbilirubinemia or hypoglycemia. Throughout childhood, the advance growth is particularly pronounced in the first year of life, after which it stabilizes with a height consistently above the 97^th ^centile between age 2 and 6 years. This childhood overgrowth may be absent (90% of the affected individuals have height and/or head circumference >2SD above the mean in Tatton-Brown series [10]). Growth in height and weight tends to normalize at puberty, probably because of the epiphysal fusion. Final height is, in the majority of cases, within the high normal range [13], particularly in females, in whom the growth pattern correlates well with the presence of advanced bone age and early puberty. Macrocephaly and large hands and feet are also consistent features [1]. Craniostenosis by premature fusion of lambdoid, sagittal and coronal sutures has been reported [11]. The reported incidence of advanced bone age in Sotos cases varies from 74% to 100% of cases, and this variability is probably related to the frequency, timing and method of assessment. This feature is present in 76% of the *NSD1*-positive individuals [10]. Bone age is often described as dysharmonic with the phalangeal age being in advance in comparison with the carpal age. X-rays of the hand are also useful for metacarpophalangeal profiles, which have been reported to produce patterns that evokes Sotos syndrome [14].

The overall craniofacial features are distinctive, especially between one and six years of age. In infancy, the face is round with disproportionate prominence of forehead and become longer in adolescence, with prominence of the pointed chin. Macrodolichocephaly, receding hairline, apparent hypertelorism with down slanting palpebral fissures, prominent jaw, malar flushing, anteverted nostrils, mild micrognathia, high arched palate and large ears are commonly identified. OFC is between the 98^th ^and 99.6^th ^centile in infancy, childhood and adulthood, and is below the 91^st ^centile in only 7% of the series of Tatton-Brown [10], 60% having an OFC greater than the 99.6^th ^percentile.

Most patients have a non progressive neurological dysfunction manifested by clumsiness and poor coordination. Cole and Hughes [3] reported a mean developmental/intelligence quotient (DQ/IQ) of 78 Sotos cases with a range 40–129 (n = 23) and, recently, de Boer *et al *[15] found that the mean IQ, in 21 individuals with *NSD1 *gene alterations was 76 (range 47–105). Delay in expressive language and motor development during the infancy is particularly common. The degree of learning disability appears extremely variable. Among the 239 *NSD1*-positive individuals in Tatton-Brown series [10], 97% presented learning difficulties. Delay in walking until after 15 months of age and speech delay until after 2.5 years are usual. Recently was reported a family with segregation of a *NSD1 *mutation with significant overgrowth but without mental deficiency [16]. Seizures are found in about 50% of patients, but in half the cases, they are febrile. Attention deficit and placid behavior may also be a component [17,18]. Psychiatric manifestations including social inhibition or psychosis have been also noticed [19,20]. Dilatation of the cerebral ventricles is common. Other non-specific neurological abnormalities include absent corpus callosum, prominent cortical sulci, cavum septum pellucidum and cavum velum interpositi [17]. Minor neuroimaging anomalies are frequently found in Sotos syndrome: prominence of the trigone in 90% of cases, prominence of the occipital horns in 75% and ventriculomegaly in 63% of cases [21,22]. Brisk deep tendon reflexes (sometimes even with a few beats of clonus) are often observed.

Variable degrees of scoliosis are present in 30% of the *NSD1*-positive cases [10]. Genu valga or vara, congenitally dislocated hips, propensity to fracturing with minimal trauma are often reported; one case of cervical instability has been reported [11,23]. Finger nails are usually deep and brittle. The incidence of congenital heart defects in Sotos syndrome is approximately 8% in the series of Cole and Hughes [3], but 21% in the series of Tatton-Brown [10]. The most common defects are septal defects and patent arterial duct. One patient is reported with Wolff-Parkinson-White pattern [24]. Recurrent upper respiratory infections and otitis media are frequent, leading to some degree of conductive hearing loss. Constipation is common and may request investigation. Genitourinary anomalies including renal anatomical anomalies (bifid, duplex or absent kidneys, vesico-ureteric reflux, pelvo-ureteric junction obstruction), cystic kidneys and genital anomalies such as hypospadius and cryptorchidism [25], are present in 15% of children with *NSD1 *abnormalities [10].

Overgrowth syndromes are often associated with neoplasms (Beckwith-Wiedemann syndrome with over-expression of *IGF-2 *and Simpson-Golabi-Behmel with *GPC3 *mutations [26]). Initial reports suggested a neoplasm frequency of 7% in Sotos patients [27]. In a survey of 224 patients with Sotos syndrome, however, the malignancy risk was found lower than 2.2% [28]. Cohen reviewed the reported neoplasias critically and suggested a tumor frequency in patients with Sotos syndrome of about 3.9% (more than in the general population) [29]. Sotos cases with *NSD1 *anomalies have been reported with small cell lung cancer [12], ganglioglioma, neuroblastoma in two cases [7,30], and acute myelocytic leukemia [31]. In the large series of Tatton-Brown, three saccrococcygeal teratomas, one presacral ganglioneuroma one neuroblastoma and one acute lymphocytic leukemia occurred in children with confirmed *NSD1 *mutation [12]. Thus, the risk of tumors in childhood appears low but the relative risk of sacrocoggygeal teratomas and neuroblastoma may be increased [10,32].

### Laboratory findings

No biochemical or endocrinological markers have been documented in patients with Sotos syndrome and/or *NSD1 *aberrations, especially endocrine and paracrine systems [33,34].

## Etiology

Although most cases are sporadic, autosomal dominant inheritance has been reported in several cases [35,36].

The report of a child with Sotos syndrome and a t(5;8)(q35;q24.1) translocation [37], has led to identification of the *NSD1 *gene, which was disrupted by the 5q35 breakpoint (Nuclear receptor SET domain containing protein) [4]. *NSD1 *intragenic mutations cause 60% to 80% of Sotos syndromes cases in Europe and USA, whereas 5q35 microdeletions encompassing *NSD1 *cause ~ 10% of cases [5-9,38]. In contrast, *NSD1 *microdeletions are the primary cause of Sotos syndrome in Japan, accounting for over 50% of cases [39-41]. Recently, partial *NSD1 *deletions were detected in Sotos cases without *NSD1 *mutation or 5q35 microdeletion [42]. The systematic *NSD1 *gene screening in a large series of provisional diagnosis of Sotos and "Sotos-like" syndrome allows to indicate that *NSD1 *is mostly found in Sotos patients. *NSD1 *abnormalities rarely occur in other childhood overgrowth phenotypes [5-7,43,44]. It is so far unknown whether *NSD1 *anomalies are involved in other entities. Current studies are focused on the identification of hotspots for *NSD1 *mutations or micro deletions and to establish a genotype-phenotype correlation. Truncating *NSD1 *mutations occured throughout the gene, but pathogenic missense mutations occurred only in functional domains [10,45]. To date, no hypermethylation or sequence abnormalities in the promotor region have been detected in Sotos patients [46].

The functions of the *NSD1 *gene have not been fully established. The gene contains 23 exons and encodes a protein of 2696 amino acids. The protein is a transcriptional intermediary factor and may act as either a nuclear receptor co-repressor or co-activator by interacting with the holo- or apoforms respectively, of the ligand binding domain of different subsets of nuclear hormone receptors. NSD1 expression in human tissue has been detected in fetal/adult brain, kidney, skeletal, muscle, spleen, lung and thymus [3]. The protein contains multiples functional domains, including the SET (Su [VAR]3–9, E [Z], trithorax) domain (initially identified in *Drosophila *genes as involved in chromatine-mediated regulation during development) and a SET associated Cys-rich (SAC) domain adjacent to the SET domain [47]. The combination of SAC and SET domains is present in proteins that function as histone-methyltransferase suggesting that NSD1 may be involved in histone modification and the establishment and maintenance of chromatin states. NSD1 contains also five plant homeodomains (PHD). The PHD domain is a zinc finger-like motif that occurs predominantly in proteins that functions at the chromatin level. NSD1 contains two PWWP (Proline-tryptophane- tryptophane-proline) domains. Their role has not been established but they are presumably involved in protein-protein interactions. Finally, NSD1 contains two distinct nuclear receptor interaction domains [4].

Rare cases of Sotos syndrome have been reported in association with other chromosomal abnormalities: apparently balanced t(3;6) (p21;p21), deletion 15q12, 45, XY, t(15q;15q) robertsonien translocation [48,49].

Seven to 35% of Sotos patients of the reported series do not have any *NSD1 *anomalies. In these patients, the involvement of the *NSD2 *and *NSD3 *genes has been already excluded by sequencing [50].

## Diagnosis methods

Diagnosis is based on clinical exam and radiographic bone age (cf. clinical description). The clinical diagnosis can be confirmed by FISH analysis or, if available, by MLPA (Multiplex Ligation Dependent Probe Amplification), a simple and reliable technique to detect 5q35 microdeletion and partial *NSD1 *deletions (both account for approximately 15% of the Sotos cases in West Europe [51]); DNA analysis by sequencing will let to look for *NSD1 *mutations.

In patients without *NSD1 *anomalies, cytogenetic studies as high resolution karyotyping and comparative genomic hybridization on microarray techniques may be useful.

## Differential diagnosis

Sotos syndrome belongs to a group of overgrowth syndromes that have some clinical features in common such as pre- and/or postnatal overgrowth and/or advanced bone age. Many of these syndromes, however, can be easily excluded on the bases of other major clinical features. The entities that should be considered in the general differential diagnosis of Sotos syndrome are: Weaver syndrome; Beckwith-Wiedemann syndrome; Perlman syndrome; Simpson-Golabi-Behmel syndrome; Fragile X syndrome; Bannayan-Zonana syndrome; *PTEN *mutations; Trisomy 15q26-qter; Nevo syndrome; Neurofibromatosis I; Marshall syndrome; Marfan syndrome; Homocystinuria; Acromegaly.

## Genetic counseling

Sotos syndrome is caused by hemizygous mutations in an autosomal non-imprinted gene. The mental difficulties in Sotos syndrome are usually in the mild-to-moderate range, and are not sufficient to account for the extreme paucity of familial Sotos cases. Fifteen familial cases of Sotos syndrome have been reported with *NSD1 *mutations [10,52,53]. It has been suggested that this lack of family cases is associated with an underlying defect in fertility associated with *NSD1 *mutations. A three generation family with *NSD1 *mutation has been reported [16].

Chromosome analysis of both parent needs to be performed when a deletion of *NSD1 *is detected in the affected child. The recurrence risk for a family with an affected child is very low (<1%). Germline mosaicism has never been reported.

The identification of *NSD1 *has been a major step in the history of Sotos syndrome and has opened a new area in the elucidation of this condition. The evaluation of the tumor frequency in patients with *NSD1 *anomalies will be important to further define the tumor risk in patients with Sotos syndrome.

## Management and outcome

In the neonatal period, phototherapy is often required because of jaundice. Sucking and swallowing difficulties need adjustments of baby food. Alternate methods of feeding may need to be considered such as the use of a nasogastric tube. Another common problem is gastroesophageal reflux which causes heartburn, vomiting, esophageal irritation and respiratory problems. Treatments may include adaptations such as eating upright and elevating the head of the bed, medications and, in rare cases, surgery. Oral-motor treatment plan may be a part of the child's therapy program.

Initial systematic screening include renal, cardiac and orthopedic clinical examination and investigations. Scoliosis and vesico-ureteral reflux have to be detected early.

In infancy and the early years, general and specialized pediatrics follow-up, every one or two year, is still important because of the possible clinical events, including constipation, respiratory infections, seizures and because of the possible increased risk of tumors. Anesthesia may require special precautions [54].

Early in the childhood, programs including infant stimulation, occupational therapy, speech therapy, and adaptive physical education play a significant role in the nurturing of a child with Sotos syndrome. Later, some children participate in regular classrooms with support and some others are enrolled in classes for appropriate education.

As mentioned previously, some Sotos patients develop behavior problems during the school age years. As a result, parents and school staff may have to spend significant amount of time and attention dealing with these problems because they can interfere with learning, social interaction and family functioning. A psychologist may work with parents and teachers to develop appropriate and effective methods.

Final body height is difficult to predict but growth in height tends to normalize after puberty.
